# Cancer-related cognitive impairment in patients with hematologic malignancies after CAR T cell therapy: a systematic review and meta-analysis of prevalence

**DOI:** 10.1007/s00520-025-09356-2

**Published:** 2025-03-22

**Authors:** Mu-Hsing Ho, Denise Shuk Ting Cheung, Tongyao Wang, Lizhen Wang, Justin Wei Ho Wong, Chia-Chin Lin

**Affiliations:** 1 School of Nursing, Li Ka Shing Faculty of Medicine, The University of Hong Kong, Pokfulam, Hong Kong, Hong Kong SAR; 2 Alice Ho Miu Ling Nethersole Charity Foundation, Tai Po, New Territories Hong Kong

**Keywords:** Cancer-related cognitive impairment, Chimeric antigen receptor T cell therapy, CAR T cell, Hematologic malignancies, Immunotherapy, Neurotoxicity

## Abstract

**Purpose:**

Cancer-related cognitive impairment is one of the symptoms of neurotoxicity among patients receiving chimeric antigen receptor (CAR) T cell therapy. Evidence of the overall estimated prevalence of cancer-related cognitive impairment following CAR T-cell therapy among patients with hematologic malignancies at short-term and long-term follow-ups is lacking. This review aimed to summarize the cognitive functioning status and estimate the prevalence of cancer-related cognitive impairment at follow-up within 1 month, 1 to 12 months, and > 12 months after CAR T cell therapy.

**Methods:**

PubMed, Cochrane Library, EMBASE, CINAHL Plus, Web of Science, and PsycINFO via ProQuest from inception through August 2024. Studies that reported on cognitive impairment among patients receiving CAR T cell therapy with valid measures were included. Data on cognitive impairment prevalence were pooled using a random-effects model.

**Results:**

In total, 16 studies involving 1407 patients were included. The pooled cancer-related cognitive impairment prevalence rates assessed using neuropsychological tests at the follow-up timepoints (< 1 month, 1–12 months, and > 12 months) were 24% [95% prediction interval (PI) 16–33%], 33% (95%, PI 9–64%), and 35% (95%, PI 23–48%), respectively. The prevalence estimates assessed using other measures were ranging from 4 to 38% across different timepoints. The leave-one-out meta-analyses quantified the impact of these potential outliers on the estimation of the overall prevalence.

**Conclusions:**

The findings stress the importance of developing targeted interventions to prevent or manage cognitive impairment in cancer patients during both short-term and long-term follow-up periods. This review also highlights the need for further research in this area to improve our understanding of the disease mechanisms and implement preventive strategies for managing cancer-related cognitive impairment.

**Supplementary Information:**

The online version contains supplementary material available at 10.1007/s00520-025-09356-2.

## Introduction

Cancer-related cognitive impairment (CRCI) refers to declines in cognitive function that related to a cancer diagnosis or experience during or after cancer treatment. These changes can include difficulty with memory, concentration, and problem-solving, and it may persist for months or years after cancer treatment [1]. The estimated prevalence of CRCI varies, with studies reported that up to 75% of cancer patients may experience some degree of cognitive problems [2, 3]. In hematologic cancer survivors particularly, the post-treatment cognitive complaints were prevalent as high as 70.6%, indicating a significant impact on cognitive function in this population [4].

Growing recognition of the intricate and multi-dimensional nature of CRCI has led to research on the etiology and underlying mechanisms. This includes examining the individual impacts of tumor burden and diverse cancer treatments, such as surgery, chemotherapy, radiation therapy, and endocrine therapy [3]. Evidence also suggested that immunotherapy, a form of cancer therapy that functions by eliciting a response from the body’s immune system to target malignant cells for destruction, may be associated with cognitive changes in some patients [5]. However, the specific nature and extent of these changes are not yet well understood. Some studies have reported that patients receiving immunotherapy may experience symptoms such as confusion, memory problems, and difficulty with attention and concentration [5, 6]. The potential mechanisms include brain microglial activation while microglia are activated in response to brain injuries and immunological stimuli and increased peripheral inflammatory cytokines crossing the blood–brain barrier [1]. It is important to note that the risk of cognitive changes associated with cancer treatment, including immunotherapy, can vary widely among patients, and the factors that contribute to these changes are not yet fully discovered.

Chimeric antigen receptor T (CAR T) cell therapy is a relatively new, effective immunotherapy that uses genetically modified T cells to target tumor cells selectively, making it more effective than chemotherapy and other immunotherapies [7]. This groundbreaking therapy has established itself as the standard of care for specific cancers, including relapsed non-Hodgkin’s lymphoma, relapsed or refractory acute lymphoblastic leukemia, and relapsed refractory multiple myeloma. Research indicates an impressive 80% complete response rate to the treatment [8]. However, common adverse events of CAR T cell therapy include cytokine release syndrome (CRS) and immune effector cell-associated neurotoxicity syndrome (ICANS), which can cause symptoms such as fever, delirium, confusion, and cognitive dysfunction, including attentional deficits, memory deficits, and executive dysfunction [9]. ICANS is a phenomenon that occurs after CAR T cell infusion and is distinct from CRS due to its clinical manifestations, timing, and response to intervention [10]. The onset of CRS and ICANS typically occurs within the first 2 weeks after infusion and usually resolves within 1 to 2 weeks [11, 12]. However, non-specific symptoms may still occur, and it can be difficult to distinguish between ICANS and other factors, such as intercurrent illness and infection. While patients are closely monitored for CRS and ICANS, CRCI are not well characterized. Given that the symptoms of ICANS appear acutely, typically within days of treatment, and the symptoms of CRCI develop more gradually and can persist for months or years after treatment, affecting memory, attention, and executive function, it is important to have a detailed understanding of these deficits to diagnose and treat them appropriately and to provide accurate prognostication [9, 11].

Clinical guidelines recommend using physical exams, history-taking, and the immune effector cell (ICE) score to assess ICANS severity. The ICE score is derived from five tasks evaluating orientation, naming, writing, attention, and command-following [13]. However, the ICE score has limitations as it lacks sensitivity to CRCI and does not provide a detailed assessment of cognition. It also has a limited dynamic range and may be subject to practice effects, as patients and caregivers may practice the items. CRCI is commonly assessed using subjective measures, including patient-reported outcomes and perceived cognitive function, while objective measures involve standardized neuropsychological tests that assess cognitive performance. Patients often describe CRCI as a subtle feeling of cognitive decline, which can negatively impact an individual’s everyday life [14]; however, subjective measures often do not correlate well with objective measures [15]. Therefore, both measures are important and should be considered when capturing CRCI. Several individuals have disclosed enduring CRCI for as long as 12 months following the administration of CAR T cell therapy [10, 12], highlighting the need for more comprehensive investigations into CRCI both immediately after treatment and in the long-term. Therefore, a more detailed understanding of CRCI associated with CAR T cell therapy is required for accurate diagnosis, treatment, and prognosis.

This systematic review and proportional meta-analysis aimed to investigate the cognitive outcomes assessed through various subjective and objective measures of patients treated with CAR T cell therapy, both in the short-term and long-term. This is important as CRCI can have adverse effects on a patient’s quality of life, and understanding the neurocognitive outcomes can help with recognizing and addressing CRCI, as well as guiding recommendations for rehabilitation and disease monitoring [1]. The review also identifies gaps in the current literature and discusses limitations to guide future research in this area.

## Methods

### Design

The guidelines including Meta-Analysis of Observational Studies in Epidemiology (MOOSE) and Preferred Reporting Items for Systematic reviews and Meta-Analyses (PRISMA) were followed in this systematic review and proportional meta-analysis. The review registration number on PROSPERO is CRD42024581909.

### Search strategy

A comprehensive search was conducted across multiple databases such as PubMed, Cochrane Library, CINAHL Plus, EMBASE, PsycINFO via ProQuest, and Web of Science. The search was performed using Medical Subject Headings (MeSH) terms and relevant keywords such as cognitive dysfunction and intensive care units and included all articles from the inception of the databases up to August 2024. The complete search strategy is outlined in Supplementary Table S1. Additionally, a manual citation chasing was performed to identify any relevant articles that were eligible.

### Eligible criteria

Inclusion criteria for the studies were as follows: (1) observational studies with retrospective and prospective cohort design reporting on cognitive functioning outcome; (2) patient population consisting of patients with hematologic malignancies who received CAR T cell therapy; and (3) identification of cognitive impairment using validated measures and available to define cognitive impairment or diagnosis by clinicians. In the case of multiple articles utilizing data from the same patient source, the study with the most comprehensive information or the largest sample size was included to avoid overcounting the prevalence of CRCI. Exclusion criteria were as follows: (1) editorials, reviews, letters to the editor, discussion paper, or conference abstracts with insufficient details on reporting cognitive functioning outcome; (2) failure to provide an operational definition of cognitive impairment; (3) observation of cognitive impairment prevalence before receiving CAR T cell therapy without post-treatment follow-up observations; or (4) publication not in English language.

### Study selection and data extraction

The title and abstract of all records identified in the literature search were independently screened by three reviewers (MHH, LW, and JWHW) who are experienced in conducting systematic reviews. Remaining articles’ full texts were independently reviewed by the same three reviewers. Each article was assessed by at least two reviewers independently. To ensure consistency and accuracy, we created a data extraction sheet in a standardized format for reviewers to record study details such as publication information (first author, year of publication, and sample size), country, and study design, where the study was conducted, cognitive functioning measures and cognitive functioning findings, as well as sample characteristics including mean age, percentage in male, and the number of cases developed CRCI at post-treatment timepoints. The information was extracted independently and reviewed for consistency and accuracy. The research team reach a consensus through discussion if there were any discrepancies.

### Risk of bias assessment

Two reviewers (MHH and LW) assessed the risk of bias of included studies using the Newcastle–Ottawa Scale [16]. This scale comprises three sections, which assess sample selection, comparability of study groups, and exposure/outcome assessment. Assessment criteria were detailed in Supplementary Table S2. The final decision of the studies—inclusion and exclusion—was made through discussion with the research team.

### Data synthesis and analysis

The Freeman–Tukey double arcsine transformation of proportions was adopted to pool prevalence estimates in this proportional meta-analysis [17]. The follow-up timepoints for the cognitive function assessments were varied, and several studies have reported multiple follow-ups across time; thus, the follow-up timepoints were categorized into three timepoints [< 1 month, 1–12 months, and > 12 months] for understanding the prevalence of CRCI in a short-term and long-term period after CAR T cell therapy. Given the diversity of patients undergoing various treatment procedures and diagnoses, as well as the inherent heterogeneity of prevalence data, we anticipated substantial between-study heterogeneity. Therefore, this proportional meta-analysis was performed using a random-effects model [18]. In situations where multiple assessment tools were used to measure cognitive impairment, prevalence data were obtained for meta-analysis from the most frequently used assessment tools. The pooled prevalence estimates are expressed as percentages, along with 95% prediction intervals (PIs). Cochran’s *Q* test and *I*^2^ statistics were used to assess statistical heterogeneity, where *I*^2^ values of 75% suggested high heterogeneity [19]. To evaluate the robustness of the pooled prevalence estimates, we conducted sensitivity analyses by excluding studies. We performed leave-one-out analyses to determine the impact of outliers [20]. A significant level of 0.05 was set. All statistical analyses were conducted using Stata 18 software.

## Results

### Systematic search and study characteristics

**Fig. 1:**
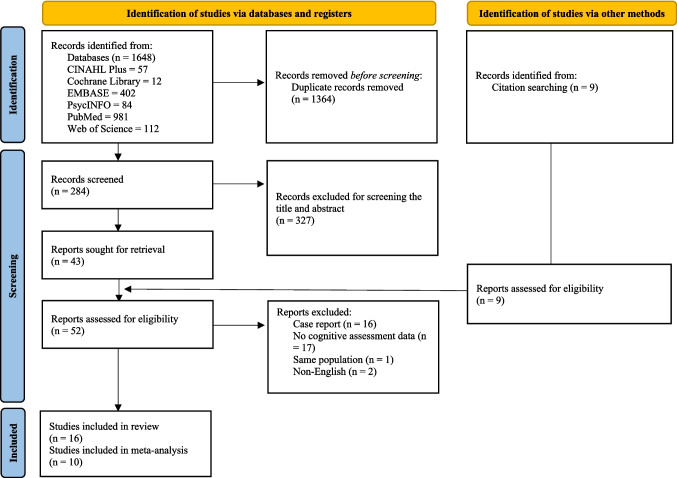
PRISMA 2020 flow diagram

**Fig. 2:**
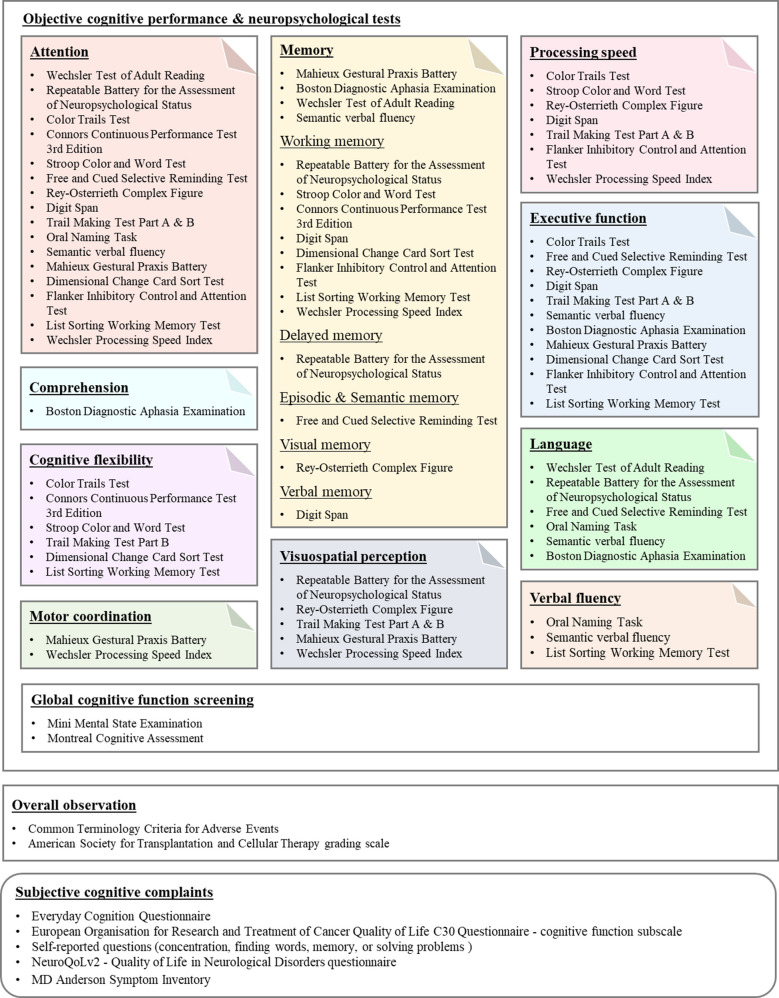
Summary of cognitive function assessments. Cancer-related cognitive assessments for patients with hematologic malignancies receiving CAR T-cell therapy

The initial search produced a total of 1648 records from all databases, which 1364 records were retained following the elimination of duplicates. From these, 16 studies that were deemed eligible according to the pre-specified inclusion criteria, and had been published between 2018 and 2024, were ultimately selected for inclusion in this review (Fig. 1). Of all studies, only one study adopted retrospective design (75.9%) [21], while the rest used a prospective cohort design (*n* = 15, 17.2%) [22–36] for reporting the quantitative results. Most studies were conducted in North America (*n* = 9, 56.3%), followed by Europe (*n* = 3, 18.8%), Asia (*n* = 1, 6.3), and Oceania (*n* = 1, 6.3%). Two studies were multicenter, prospective research recruiting participants internationally [24, 25]. Several cognitive functioning assessments were used including objective and subjective measures. For objective measures, neuropsychological assessments and overall observations as well as cognitive function screening such as Common Terminology Criteria for Adverse Events (CTCAE), American Society for Transplantation and Cellular Therapy (ASTCT) grading scale, Mini Mental State Examination (MMSE), and Montreal Cognitive Assessment (MoCA). Subjective measures such as Everyday Cognition Questionnaire (ECog), European Organisation for Research and Treatment of Cancer Quality of Life C30 Questionnaire (EORTC QLQ-C30) cognitive function subscale, Quality of Life in Neurological Disorders questionnaire (NeuroQoLv2), and MD Anderson Symptom Inventory (MDASI) were used to capture perceived, subtle cognitive changes. A detailed summary of all cognitive function assessments used in the included studies was displayed in Fig. 2. Figure 2 also categorized all neuropsychological tests, and their corresponding cognitive functions domains measured for. A total of 1407 patients were included, with 53–80% of these patients were men. Table 1 shows the key characteristics of the included studies.

**Table 1:** Characteristics of included studies (*n* = 16)

Author, year, country	Sample size	Diagnosis	Agent/drug/ of CAR-T therapy	Mean age	Male (%)	CRCI measurement	Cognitive functioning results
Barata, [22], USA	118	102 LBCL, 16 other hematologic malignancies	Axicabtagene ciloleucel, Tisagenlecleucel, Brexucabtagene autoleucel	61	59	Everyday Cognition Questionnaire	No significant mean level changes from baseline to day 90 From day 90–360, participants reported: - Worsening global cognition (*p* = .01, *d* = .18) - Memory (*p* = .04, *d* = .15) - Language (*p* = .04, *d* = .15) - Organization (*p* = .03, *d* = .17) - Divided attention (*p* = .001, *d* = .28)
Belin, [23], France	84	Relapsing BCL (76 DLBCL, 6 tFL, 2 FL)	Axicabtasene ciloleucel, Tisagenlecleucel	59 (median)	69	CTCAE, ASTCT grading scale, and clinical notes assessed by neurologists	Frequencies: 10 aphasia, 7 executive disorders, 5 agraphia, 5 cognitive slowness, 2 apraxia, 2 disorientation, 2 restlessness, 1 attention, 1 dysarthria, 1 dyscalculia, 1 hemineglect syndrome, 1 memory disorders, 1 hallucination - Mean duration of all neurological (including cognitive) symptoms was 6 days
Cohen, [24], USA and Japan	97	Relapsed or refractory multiple myeloma	Ciltacabtagene autoleucel	61 (median)	59	CTCAE, ASTCT grading scale, patient reported and clinician-assessed	ICANS was observed in 16% of patients; most patients had grade 1/2 ICANS, one patient had grade 3 and another had grade 4 ICANS (including cognitive symptoms) - Median onset was 8 days, and mean duration was 4 days
Delforge, [26], USA	128	Relapsed and refractory multiple myeloma	Idecabtagene vicleuce	NR	NR	EORTC QLQ-C30 cognitive functioning	Improvements across assessment visits (months 2–9) but these improvements did not persist through months 12–18
Delforge, [25], International^a^	386	Relapsed or refractory multiple myeloma	Idecabtagene vicleucel and standard regimens	63 (median)	61	EORTC QLQ-C30 cognitive functioning	Improvements across assessment visits (baseline to month 20) but did not exceed the within-group minimally important difference thresholds
Hoogland, [27], USA	117	NHL: 75 DLBCL, 39 FL/tFL, 2 PMBL, 1 MCL	Axicabtagene ciloleucel, Tisagenlecleucel	60.9	62	WTAR, RBANS, CTT-1 & 2, CPT3, SCWT	Total neurocognitive performance and executive function declined from baseline to day 90 and then improved from day 90 to day 360 (*p* < .04) - The magnitude of improvement from baseline to day 360 was small (*d* = .16 in total neurocognitive performance and *d* = .22 in executive function) - Small linear declines in visuospatial abilities over time (*d* = − .26, *p* = .03)
Levine, [28], USA	137	FL/tFL	Tisagenlecleucel	12 (median)	53	CTCAE	Frequencies: 13 confusion, 4 mental status changes, 3 cognitive disorder
Li, [29], China	29	PMBL, MCL	CD19 targeted CAR T cells	62 (median)	58.6	CTCAE	Percentages: confusion (10.3%) and aphasia (6.8%)
Maillet, [30], France	27	Relapsing B-cell lymphoma	Axicabtagene ciloleucel. Tisagenlecleucel	58	59	MMSE, French FCSRT, Recall ROCF, DGS, TMT Part A&B, Stroop test, oral naming task of 80 images, sVF, dictation and repetition of BDAE, Praxis battery, QMRP	Performances improved between baseline and follow-up: - Visuo-construction (*p* < .001) - Visuospatial memory (*p* < .001) - Working memory (*p* = .002) 30% of the sample reported memory complaints at baseline compared to 11% at follow-up
Möhn, [31], France	15	10 DLBCL, 5 tFL	Tisagenlecleucel	56.3	60	MoCA	MoCA scores remained in the normal range for 11 of the 15 patients within 10-day monitoring period - ICANS was diagnosed in the remaining 4 patients, and 3 of these patients showed steep drop in MoCA results. Two patients MoCA scores improved to the normal range
Ruark, [32], USA	40	15 Relapse or refractory chronic lymphocytic leukemia, 11 ALL, 14 NHL: 7 DLBCL, 1 HGBCL, 1 Burkitt lymphoma, 3 MCL, 2 FL	Biological: autologous anti-CD19CAR-4-1BB-CD3zeta-EGFRt-expressing T lymphocytes	54 (median)	62.5	4 self-report cognitive functioning questions (concentration, finding words, memory, or solving problems)	37.5% of patients reported one or more cognitive difficulties including: - Memory (35%) - Word finding (30%) - Concentration (22.5%) - Problem-solving (12.5%) - 10% of patients reported experiencing all four cognitive difficulties
Sales, [33], Australia	53	Relapsed DLBCL multiple myeloma or mediastinal lymphoma	Axicabtagene ciloleucel, Tisagenlecleucel	NR	NR	MoCA, Structured standard neurological examination	Changes in cognition were manifested in most patients, with a more substantial drop noted in their MoCA compared to ICE scores - All manifestations of neurotoxicity were short-lived and resolved within a 1-month period with a mean duration of 8.2 days (range = 1–33)
Shalabi, [34], US	22	Relapsed or refractory B cell malignancies	CD22 targeted CAR T cells	17.9	63.9	NIH Toolbox: DCCS, FICAT, LSWMT, Wechsler PSI	No significant difference between pre- and post-infusion on the DCCS, FICAT, and LSWMT at the group level - There was a significant improvement in PSI (mean difference = 6.06, *t* = 2.15, *p* = .048)
Sidana, [35], USA	34	8 Myeloma, 23 lymphoma, 3 leukemias or other myeloid neoplasms	NR	62 (median)	53	NeuroQoLv2	Significant improvement in NeuroQoLv2 score at month 4 compared to baseline - However, this improvement was not clinically meaningful (change < 8 points) and no significant difference at the other time points
Wang, [36], USA	60	3 acute leukemia, 57 LBCL	Axicabtagene ciloleucel, Tisagenlecleucel	56.4	76.7	MDASI (one item on cognition/difficulty remembering)	No significant difference in the cognition item ratings on the MDASI between patients who received CAR T less than 30 day, between 30 and 90 days, and more than 90 days of completing the study
Wudhikarn, [21], USA^b^	60	35 De novo DLBCL, 25 TIL	Axicabtagene ciloleucel, Tisagenlecleucel	63 (median)	80	CTCAE, ASTCT grading scale	Common neurologic events: confusion/cognitive impairment (*n* = 11), aphasia (*n* = 3). Median onset for ICANS was 5 days

^a^Belgium, Canada, France, Germany, Italy, Japan, The Netherlands, Norway, Spain, Switzerland, UK, USA. ^b^Retrospective design, the rest studies adopted prospective design

*CAR-T*, chimeric antigen receptor T cell; *CRCI*, cancer-related cognitive impairment; *LBCL*, large B cell lymphomas; *DLBCL*, diffuse large B cell lymphoma; *tFL*, transformed follicular lymphoma; *NHL*, non-Hodgkin lymphoma; *PMBL*, primary mediastinal B cell lymphoma; *MCL*, mantle cell lymphoma; *ALL*, acute lymphoblastic leukemia; *HGBCL*, high-grade B cell lymphoma; *TIL*, transformed indolent lymphoma; *NR*, not reported; *CTCAE*, Common Terminology Criteria for Adverse Events; *ASTCT*, American Society for Transplantation and Cellular Therapy; *ICANS*, immune effector cell-associated neurotoxicity syndrome; *EORTC QLQ-C30*, European Organisation for Research and Treatment of Cancer Quality of Life C30 Questionnaire; *WTAR*, Wechsler Test of Adult Reading; *RBANS*, repeatable battery for the assessment of neuropsychological status; *CTT*, color trails test; *CPT3*, Connors Continuous Performance Test Third Edition; *SCWT*, Stroop Color and Word Test; *MMSE*, Mini Mental State Examination; *FCSRT*, Free and Cued Selective Reminding Test; *ROCF*, Rey-Osterrieth complex figure; *DGS*, digit span; *TMT*, Trail Making Test; *sVF*, semantic verbal fluency; *BDAE*, Boston Diagnostic Aphasia Examination; *QMRP*, Prospective and Retrospective Memory Questionnaire; *MoCA*, Montreal Cognitive Assessment; *NIH*, National Institutes of Health; *DCCS*, Dimensional Change Card Sort Test; *FICAT*, Flanker Inhibitory Control and Attention Test; *LSWMT*, List Sorting Working Memory Test; *PSI*, Processing Speed Index; *NeuroQoLv2*, Quality of Life in Neurological Disorders questionnaire; *ICE*, immune effector cell-associated encephalopathy; *MDASI*, MD Anderson Symptom Invento

### Risk of bias assessment

The risk of bias assessment result is summarized in Supplementary Table S2. The methodological quality of all studies deemed satisfactory, and no study was excluded due to the risk of bias assessment. Most studies were rated as having good quality; the variations in scores were primarily attributed to the absence or ambiguity of adjustments made for confounding factors.

### Cognitive functioning status and risk factors reported in the included studies

There were discrepancies between included studies while some reported improved or declined cognitive functioning within a month, within a year and more than 1 year after CAR T cell infusion. The main findings of cognitive functioning were presented in Table 1. Of note, only very limited studies reported risk factors of CRCI among patients who received post-CART T cell therapy. Demographic factors such as sex, length of hospital stay, ICANS grade, CRS grade, and number of lines of previous therapy were not significantly associated with CRCI [22, 27, 32]. In addition, two studies reported that age was not associated with CRCI among adult patients [22, 27]. Nevertheless, Shalabi et al. [34] documented that three out of the eight adult patients exhibited reduced cognitive performance in at least one cognitive domain while all seven pediatric patients evaluated (aged ≤ 18 years) did not exhibit declines (defined as a 0.75 standard deviation change from baseline) in any of the four cognitive domains analyzed including attention, memory, processing speed, and cognitive flexibility.

### Prevalence of cancer-related cognitive impairment

**Fig. 3:**
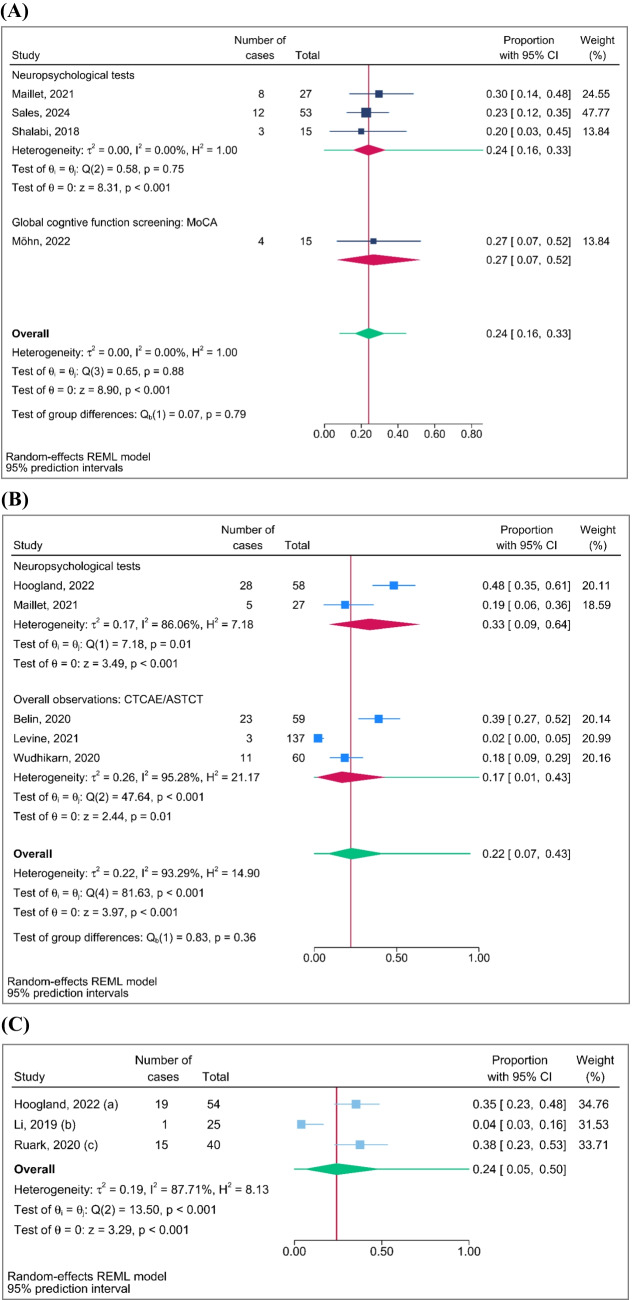
Pooled proportions of cancer-related cognitive impairment at **A** within a month, **B** 1 to 12 month(s), and **C** over 12-month follow-up. (a) Neuropsychological tests. (b) Overall observations: CTCAE. (c) Subjective cognitive complaints (self-reported questions)

**Fig. 4:**
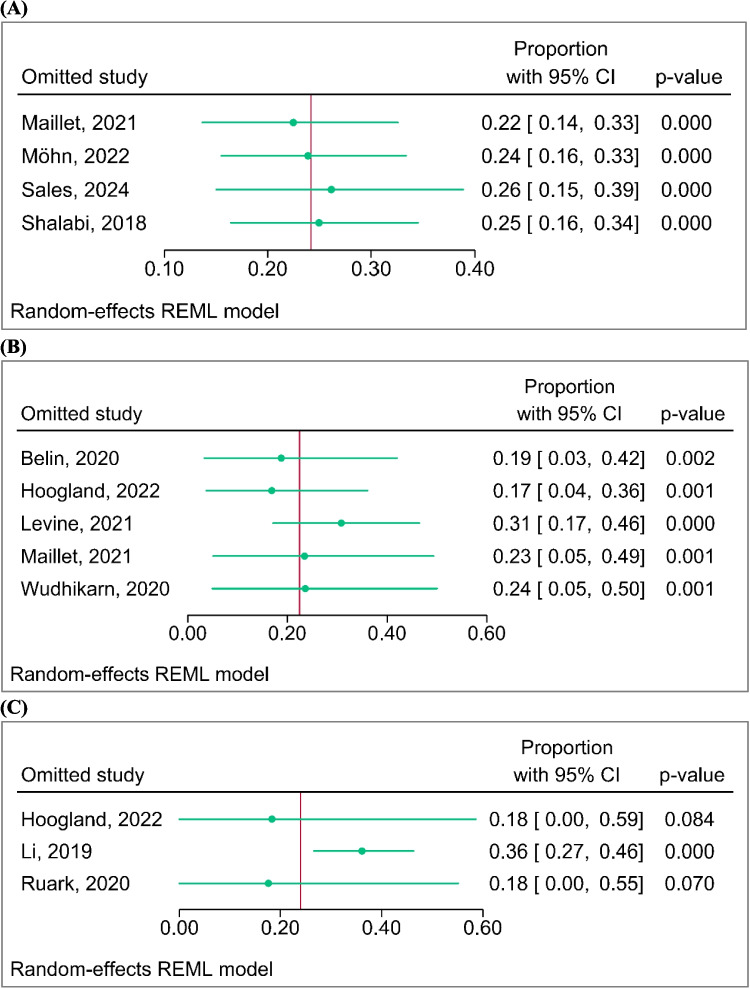
Leave-one-out meta-analysis on cancer-related cognitive impairment prevalence at **A** within a month, **B** 1 to 12 month(s), and **C** over 12-month follow-up

The pooled prevalence rates of CRCI at the follow-up timepoints < 1 month, 1–12 months, and > 12 months were reported. At the follow-up timepoint of less than 1 month, the pooled prevalence rate of CRCI assessed using neuropsychological tests was 24% (95%, PI 16–33%, *n* = 3). One study assessed CRCI using global cognitive function screening (MoCA), and the prevalence rate was 27% (95%, PI 7–52%) (Fig. 3A). At the follow-up timepoint of 1 to 12 months, the pooled prevalence rate of CRCI assessed using neuropsychological tests was 33% (95%, PI 9–64%, *n* = 2), while three studies using overall observations (CTCAE/ASTCT) reported a prevalence rate of 17% (95%, PI 1–43%) (Fig. 3B). At the follow-up timepoint of more than 12 months, the prevalence rate of CRCI assessed using neuropsychological tests was 35% (95%, PI 23–48%, *n* = 1). One study using overall observations (CTCAE/ASTCT) reported a prevalence rate of 4% (95%, PI 3–16%), and another study that used self-reported questions to assess subjective cognitive complaints reported a prevalence rate of 38% (95%, PI 23–53%) (Fig. 3C). Leave-one-out analyses addressed identified outliers and influential studies and demonstrated that excluding single study at a time yielded increased and decreased prevalence rates of CRCI (Fig. 4). As shown in Fig. 4A, the prevalence of CRCI at the follow-up timepoints less than 1 month dropped to 22% when excluding study conducted by Maillet et al. [30]. For the follow-up timepoints at 1–12 month(s), the omission of study (3) [28] or (2) [27] seems to have a relatively larger influence when compared with other studies on the estimation of the overall prevalence. Omitting study (1) causes the overall prevalence to increase by roughly 9%, whereas omitting study (2) causes the overall risk ratio to decrease by roughly 5% (Fig. 4B). For the follow-up timepoints more than 12 months, the prevalence of CRCI increased dramatically to 36% when excluding study conducted by Li et al. [29] (Fig. 4C). These sensitivity analysis results indicate the prevalence rates are varying with potential outliers. The leave-one-out meta-analysis shown in Fig. 4 quantified the impact of these potential outliers on the estimation of the overall prevalence. Therefore, these overall results with potential outliers need to be interpreted with caution.

## Discussion

This systematic review and proportional meta-analysis summarized the cognitive functioning status and estimated the prevalence of CRCI following CAR T cell therapy at follow-up within 1 month, 1 to 12 months, and > 12 months. The systematic review found 16 studies involving 1648 patients. The majority of these studies were considered to have a low risk of bias according to the NOS criteria. The findings suggest that CRCI is prevalent among patients receiving CAR T cell therapy, especially during the initial follow-up period within 1 month. This is the first proportional meta-analysis to report the prevalence of CRCI among patients with hematologic malignancies who received CAR T cell therapy. The results indicated that the CRCI prevalence rate assessed using neuropsychological tests can be as high as 24% within a month after CAR T cell therapy. For those patients who received CAR T cell therapy for more than 12 months, the estimated prevalence assessed using neuropsychological tests was 35%, indicating that one-third of the patients developed CRCI at the long-term follow-up assessment. This study adds to the existing knowledge by providing estimates of the prevalence of CRCI after CAR T cell therapy at short-term and long-term follow-ups. Nevertheless, the results of this meta‐analysis must be interpreted with caution due to the high between‐ study heterogeneity.

### Cognitive function in patients who received CAR T cell therapy

This review found that there were discrepancies in the included studies while some reported of maintained, improved, or declined cognitive function within a month, within a year, and more than 1 year following CAR T cell therapy. However, noted that these studies were adopted different cognitive function measures (i.e., subjective and objective approaches) and the heterogeneity of study population (i.e., cancer severity, treatment received, baseline cognitive condition, CAR T cell therapy agent type, and history of other diseases that may influence the cognitive function), it is difficult to estimate the incidence of newly developed neurotoxicity symptoms or trajectories of CRCI in this particular group of patients. These varied outcomes may be indicative of the diversity within the study population. Subsequent research efforts should aim to explore the potential impact of these factors on cognitive function.

We aimed to investigate the cognitive functioning status observed in both short-term and long-term follow-ups. Despite some observed patterns, discerning cognitive deficits has proven difficult due to the heterogeneity of the studies. The review highlights the common occurrence of neurotoxicity related to CAR T cell therapy and CRCI in hematological malignancies. These impairments range from attention fluctuations to deficits in memory, executive function, and visuospatial construction [21, 22, 27, 30, 32, 33]. Furthermore, specific deficits on certain cognitive functioning domains such as executive function, language, and disorientation have been noted, particularly in the short-term follow-up period, but the reason for this commonality remains unclear [21, 23]. The review stresses the need for future studies to conduct longitudinal analyses of cognition in both acute and chronic settings of CAR T cell therapy. This evidence is essential as it enhances our comprehension of disease mechanisms and equips us with the necessary tools to monitor and implement suitable preventive strategies or nonpharmacological interventions for CRCI.

### Cancer-related cognitive impairment assessments in patients received CAR T cell therapy

This review emphasizes the necessity of combining neuropsychological tests and self-reported assessment to effectively characterize cognitive functioning status in this specific patient group. For instance, individuals who are unaware of their cognitive shortcomings may downplay deficits that are noticeable during assessments [37]. On the other hand, certain subjective cognitive impairments may not be detectable through neuropsychological assessments. Since cognitive tests have not been specifically validated for individuals undergoing CAR T cell therapy, there is a possibility that these tests may lack sensitivity or validity within this particular patient group [38]. In the broader cancer literature, attempts were undertaken to validate neuropsychological tests for assessing CRCI. The International Cognition and Cancer Task Force (ICCTF) has provided clear recommendations for the use of neuropsychological tests in assessing cognitive impairment [39]. These tests are specifically designed to evaluate cognitive function in cancer patients and include measures of attention, memory, executive function, processing speed, and language [1, 39]. The use of these tests can provide a more comprehensive assessment of cognitive impairment compared to the ICE score, which has limitations. By using these neuropsychological tests, healthcare providers can more accurately diagnose and treat cognitive impairment in patients who have undergone CAR T cell therapy or other cancer treatments.

Among all included studies, only two studies [27, 30] highlighted that the ICCTF recommendations were followed, stressing the need for future studies to use such tests to assess CRCI in this particular population. Apart from utilizing a core set of neuropsychological tests, the following recommendations by ICCTF and existing cancer literature for future research investigating CRCI in patients who have received CAR T cell therapy are as follows: (1) defining common criteria for CRCI particular in those studies using subjective measure to capture subtle change in cognition. Establish criteria for defining cognitive impairment can help ensure consistency in assessing cognitive changes across different studies; (2) incorporating longitudinal assessments for CRCI that include an assessment before CAR T cell infusion to track changes in cognitive function over time following CAR T cell therapy. This can provide insights into the course of CRCI and potential recovery patterns; (3) minimizing practice effects and measurement error is particularly important in uncontrolled trials where differential practice effects cannot be analyzed. Some included studies assessed cognitive function using objective tests or screening tool in a frequent and short period of time (e.g., daily within 10 days after CAR T cell infusion) which may cause potential practice effects or memory bias; (4) collaborating across multiple institutions to collect larger samples and address research questions related to the cognitive effects of CAR T cell therapy in patients with hematologic malignancies. Multicenter studies can help provide more robust data and insights into cognitive impairment in this population [39, 40]. By incorporating these recommendations into future studies focusing on CRCI in patients with hematologic malignancies who have undergone CAR T cell therapy can enhance the understanding of CRCI associated with this treatment modality and improve patient care and outcomes in this specific population.

### Strengths and limitations

This review’s strength lies in its comprehensive literature search utilized six databases, which resulted in the inclusion of a large number of studies from diverse geographic regions for meta-analyses. Validated appraisal tool was utilized to assess the methodological quality of included studies, and none of the studies included in this meta-analysis was found to have a high risk of bias.

Although this review has several strengths, it also has certain limitations that must be taken into account. First, the significant heterogeneity was observed in our pooled analyses, which suggests that the results should be interpreted with caution. This issue is not unique to our study, as other proportional meta-analyses investigating the prevalence of a particular condition have also reported marked heterogeneity, which is reflected in their elevated *I*^2^ values [41]. In proportional meta-analysis, high *I*^2^ values are expected and may not represent important between-study heterogeneity [41]. Second, despite prevalence in subgroups were compared at the regional level, studies were mainly conducted in North America and Europe (predominately the USA), whereas Oceania and Asia populations were underrepresented. The generalizability of the prevalence estimates to other regions may be constrained due to certain limitations of this review. Thirdly, when multiple instruments were used to assess cognitive function, we selected prevalence data from the most frequently used instrument across the studies which may cause the selection bias. Fourth, other sensitivity analysis such as subgroup analyses and meta-regression are not performed due to the small number of included studies. Lastly, the exclusion of non-English articles could have led to the exclusion of pertinent studies.

## Conclusion

The present systematic review and proportional meta-analysis summarized the cognitive functioning status and pooled estimated prevalence rates of CRCI at three different follow-up timepoints: < 1 month, 1–12 months, and > 12 months. The results showed that the prevalence rates were 30%, 22%, and 24%, respectively. These findings suggest that cognitive impairment is a common complication in cancer patients across different follow-up periods. This study also highlights the need for further research to develop targeted interventions to prevent or manage CRCI at short-term and long-term follow-ups.

## Supplementary Information

Below is the link to the electronic supplementary material.

Supplementary file1 (DOCX 28.3 KB)

## Data Availability

No datasets were generated or analysed during the current study.
