# Supplementary material for: Cancer-related cognitive impairment in patients with hematologic malignancies after CAR T cell therapy: a systematic review and meta-analysis of prevalence
Source: Support Care Cancer. 2025 Mar 22;33(4):312. doi: 10.1007/s00520-025-09356-2 (PMC11929693; doi:10.1007/s00520-025-09356-2)
Supplement: Supplementary file 1 — Supplementary file1 (DOCX 28.3 KB) [file 520_2025_9356_MOESM1_ESM.docx]

Supplementary information

**Supplementary Table S1.** Full search strategy

| **Cochrane Library** | | |
| --- | --- | --- |
| ID | Search queries | Results |
| #1 | MeSH descriptor: [Cognitive Dysfunction] explode all trees | 4,093 |
| #2 | MeSH descriptor: [Neurotoxicity Syndromes] explode all trees | 1,132 |
| #3 | MeSH descriptor: [Cognition Disorders] explode all trees | 8,545 |
| #4 | (cogniti* or cognitive impairment or "cancer-related cognitive impairment" or "neurotoxic*"):ti,ab,kw | 108,494 |
| #5 | MeSH descriptor: [Immunotherapy, Adoptive] explode all trees | 8,545 |
| #6 | ("CAR T-cell therap*" or "chimeric antigen receptor T-cell" or "CAR T cell"):ti,ab,kw | 542 |
| #7 | (#1 or #2 or #3 or #4) AND (#5 or #6) | 12 |
| Trials |  | 9 |
| **PubMed** | | |
| ID | Search queries | Results |
| #1 | (Cognitive Dysfunction[MeSH Terms]) OR (Neurotoxicity Syndromes[MeSH Terms]) OR (Cognition Disorders[MeSH Terms]) | 154,530 |
| #2 | "cogniti*"[Title/Abstract] OR "neurotoxic*"[Title/Abstract] OR "cancer-related cognitive impairment"[Title/Abstract] | 637.880 |
| #3 | "Immunotherapy, Adoptive"[MeSH Terms] | 15,401 |
| #4 | "CAR T-cell therap*"[Title/Abstract] OR "chimeric antigen receptor T-cell"[Title/Abstract] OR "CAR T cell"[Title/Abstract] | 14,209 |
| #5 | (#1 or #2) AND (#3 or #4) | 981 |
| **EMBASE** | | |
| ID | Search queries | Results |
| #1 | cognitive impairment/ | 149,370 |
| #2 | cognitive defect/ | 234,863 |
| #3 | neurotoxicity/ | 119,601 |
| #4 | (cogniti* or cognitive impairment or "cancer-related cognitive impairment" or "neurotoxic*"):ti,ab,kw | 845,154 |
| #5 | chimeric antigen receptor T-cell / | 14,017 |
| #6 | ("CAR T-cell therap*" or "chimeric antigen receptor T-cell" or "CAR T cell"):ti,ab,kw | 26,814 |
| #7 | 1 or 2 or 3 or 4 | 967,206 |
| #8 | 5 or 6 | 26,981 |
| #9 | 7 and 8 | 402 |
| **PsycINFO** | | |
|  | (SU(Cogniti* or "Cognitive Impairment" or "Cognitive Dysfunction" or " Cognition Disorders) OR (Cognitive Defect or neurotoxicity or "cancer-related cognitive impairment")) AND (SU(CAR T-cell OR "CAR T cell " OR "chimeric antigen receptor T-cell" OR "chimeric antigen receptor T cell") | 84 |
| **CINAHL Plus** | | |
|  | (SU(Cogniti* or "Cognitive Impairment" or "Cognitive Dysfunction" or " Cognition Disorders) OR (Cognitive Defect or neurotoxicity or "cancer-related cognitive impairment")) AND (SU(CAR T-cell OR "CAR T cell " OR "chimeric antigen receptor T-cell" OR "chimeric antigen receptor T cell") | 57 |
| **Web of Science** | | |
|  | TS=(Cogniti* or "Cognitive Impairment" or "Cognitive Dysfunction" or " Cognition Disorders) OR (Cognitive Defect or neurotoxicity or "cancer-related cognitive impairment")) AND (SU(CAR T-cell OR "CAR T cell " OR "chimeric antigen receptor T-cell" OR "chimeric antigen receptor T cell") | 112 |

**Supplementary Table S2.** Risk of Bias Assessment According to Newcastle-Ottawa Scale

| First author, year | Selection | Comparability | Outcome | Total Score | Decision |
| --- | --- | --- | --- | --- | --- |
| Barata, 2022 | ★★★☆ | ★☆ | ★★★ | 7/9 | Included |
| Belin, 2020 | ★★★★ | ★★ | ★★★ | 9/9 | Included |
| Cohen, 2022 | ★★★☆ | ★☆ | ★★★ | 7/9 | Included |
| Delforge, 2022 | ★★★★ | ★★ | ★★★ | 9/9 | Included |
| Delforge, 2024 | ★★★★ | ★★ | ★★★ | 9/9 | Included |
| Hoogland, 2022 | ★★★★ | ★★ | ★★★ | 9/9 | Included |
| Levine, 2021 | ★★★★ | ★★ | ★★★ | 9/9 | Included |
| Li, 2019 | ★★★☆ | ★☆ | ★★★ | 7/9 | Included |
| Maillet, 2021 | ★★★☆ | ★★ | ★★★ | 8/9 | Included |
| Möhn, 2022 | ★★★★ | ★★ | ★★★ | 9/9 | Included |
| Ruark, 2020 | ★★★★ | ★★ | ★★★ | 9/9 | Included |
| Sales, 2024 | ★★★☆ | ★★ | ★★★ | 8/9 | Included |
| Shalabi, 2018 | ★★★☆ | ★☆ | ★★★ | 7/9 | Included |
| Sidana, 2022 | ★★★★ | ★★ | ★★★ | 9/9 | Included |
| Wang, 2021 | ★★★☆ | ★★ | ★★★ | 8/9 | Included |
| Wudhikarn, 2020 | ★★★☆ | ★☆ | ★★★ | 7/9 | Included |

***Note.*** Scores are 0–4 stars for selection, 0–2 stars for comparability, and 0–3 stars for outcome. Studies scored 0 or 1 star in selection domain, or 0 stars in comparability domain, or 0 or 1 stars in outcome/exposure domain were considered having poor methodological quality.
